# Cerebral Venous Thrombosis After Unintentional Dural Puncture: Raising awareness for an uncommon cause of postpartum headache

**DOI:** 10.5152/TJAR.2023.22124

**Published:** 2023-04-01

**Authors:** Ana Sousa Rodrigues, Luís Montenegro, Catarina Vieira Luz Alves, Núria Mascarenhas, Maria Patrocínio Lucas, Daniel Pedro

**Affiliations:** 1 Department of Anaesthesiology of Hospital Garcia de Orta, Almada, Portugal

**Keywords:** Cerebral venous thrombosis, neuroaxial anaesthesia, obstetric anaesthesia, postdural puncture headache, postpartum headache

## Abstract

Headache is a common symptom in the postpartum period, which can have a varied aetiology. Although rare, cerebral venous thrombosis can be a fatal complication in the parturient. Dural puncture is considered as one of the risk factors for cerebral venous thrombosis and the proposed mechanism pathogenesis can be explained by the components of Virchow’s triad: stasis of the blood, hypercoagulability, and endothelial damage. Headache is usually the most frequent symptom and can mimic those of postdural puncture headache, which can delay the diagnosis.

We will report a case of an 18-year-old woman that develops a postpartum headache after an accidental dural puncture during epidural catheter placement for labour analgesia. Our patient was initially managed for postdural puncture headache, but later the character changed, which made us look for a differential diagnosis. After a multidisciplinary approach, neuroimaging confirmed the diagnosis of cerebral venous thrombosis.

This case report emphasises the importance of a careful differential diagnosis of postpartum headache particularly if the headache persists or changes its character. Brain imaging and multidisciplinary evaluation can lead to prompt diagnosis and initiation of appropriate treatment.

**Main Points**

- Postpartum headache is a common symptom that can have a varied aetiology.
- Dural puncture is considered a risk factor for cerebral venous thrombosis.
- Cerebral venous thrombosis should be considered in the differential diagnosis of postpartum headache, especially after neuroaxial blocks.
- Multidisciplinary team management and neuroimaging are essential to an accurate diagnosis and a timely treatment.

## Introduction

Postpartum headache is one of the most common symptoms in the postpartum period. The most common types are primary headaches, like migraine and tension-type. Secondary headaches result from obstetric complications, neurologic lesions, or regional anaesthesia complications.^1^

Postdural puncture headache (PDPH) is a frequent complication after neuroaxial techniques. It occurs after spinal anaesthesia or an unintentional dural puncture as a complication of epidural anaesthesia.^2^

Cerebral venous thrombosis (CVT) is an infrequent but potentially fatal condition. The incidence of peripartum CVT is about 12 in 100 000 deliveries and a small number of cases were described after a dural puncture.^3,4^ Acute or subacute headache is the presenting feature in 70%-90% of patients; seizures and stroke deficits may follow if cortical infarction occurs.^5^ Computed tomography (CT) and magnetic resonance (MR) with venography are the preferred methods for detecting CVT. The diagnostic finding is a segment of vein without blood flow. European Stroke Organization guidelines recommend parenteral anticoagulation with low-molecular-weight heparin (LMWH) in acute CVT and decompressive surgery if brain herniation.^6^

As neuroaxial analgesia and anaesthesia are the recommended techniques in obstetric patients, anaesthesiologists are frequently asked to evaluate women with postpartum headaches. We will report a case of unintentional dural puncture during epidural labour analgesia that leads to PDPH and possibly contributed to the development of CVT. This case report was published with the consent of the patient.

## Case Presentation

An 18-year-old pregnant woman at 40 weeks of gestation was admitted for labour induction. She had a past medical history of obesity and did not have personal or family history of clotting disorder.

For treatment of first-stage labour pain, she requested epidural analgesia. During the first attempt of placement of an epidural catheter, an accidental dural puncture was noted. Successful placement of an epidural catheter was made on the second attempt. Seventeen hours after, the patient had a spontaneous vaginal delivery.

On the first day postpartum, she started to complain of frontal headaches that exacerbated in the upright position and relieved in the supine position, without other associated symptoms. A diagnosis of PDPH was made. For pain relief, oral analgesics were prescribed and an epidural blood patch was performed, with symptomatic improvement. The patient was discharged home on the next day and was advised to return to the hospital if the headache persists or if there were new neurological symptoms.

One day after discharge, the patient was readmitted with a severe non-positional frontal headache that did not relieve with oral analgesics, associated with nausea and photophobia. Further neurological examination showed no neurological deficit.

After a multidisciplinary evaluation, she underwent CT venography that revealed parietal focal acute subarachnoid haemorrhage and filling defects in the adjacent cortical vein. The MR with venography confirmed the diagnosis of CVT. The prothrombotic screen revealed Protein S deficiency and heterozygous MTHFR C677T mutation.

Anticoagulation was initiated with LMWH and then was switched to rivaroxaban by protocol. Four days after, the patient was discharged symptom-free with oral anticoagulation therapy. At a 3-month follow-up, the patient remained asymptomatic.

## Discussion

An unintentional dural puncture occurs in 0.15%-1.5% of labour epidural analgesia, and 50%-80% of these cases are followed by PDPH. Headaches normally occur in the first 72 hours after the procedure and are characterised by their postural component, which increases with sitting or standing and improves in the supine position. The typical location is occipital and/or frontal with radiation into the neck and shoulder. Symptomatic therapy includes hydration, caffeine, and simple analgesics. When this conservative management is insufficient, an autologous epidural blood patch is indicated.^7^

In our case, there was an unintentional dural puncture during an attempt of placement of an epidural catheter. Following, on the first day postpartum, the patient developed typical PDPH symptoms which improved with an epidural blood patch. Two days later, the headache intensity worsened and changed its character, which made us look for a differential diagnosis. In fact, this course of action is in agreement with the existing literature, which refers that suspicion of CVT is typically more complicated in patients with postpartum headaches that received epidural analgesia. Patients who follow this clinical course are often initially treated for PDPH. However, in CVT, the headache usually does not vary with changes in body position and is more diffuse in location. Cerebral venous thrombosis diagnosis is frequently delayed until new neurological symptoms occur or PDPH treatment fails, which results in referral for neuroimaging.^8^

Dural puncture is considered one of the risk factors for CVT. The proposed mechanism pathogenesis can be explained by the components of Virchow’s triad: stasis of the blood, hypercoagulability, and endothelial damage. First, according to the Monro–Killie doctrine, intracranial components (blood, cerebrospinal fluid (CSF), and brain) are in a state of pressure balance, and in pathological conditions, the decrease or increase of one of these elements will lead to a compensatory change in the volume of the others. In this case, loss of CSF leads to compensatory cerebral vasodilatation, which induces slowing of cerebral blood flow.^4^ Secondly, the reduction in absorption of CSF causes an increase in the blood viscosity in the venous sinuses.^9^ Thirdly, the negative spinal-cranial gradient pressure leads to a stretching of the cerebral vessels making the vascular endothelium prone to damage. This also might explain the focal acute subarachnoid haemorrhage seen in this case.

A retrospective study showed that dural puncture seems to trigger CVT, particularly in patients with predisposing disorders.^9^ The prothrombotic screen of our patient revealed Protein S deficiency and heterozygous MTHFR C677T mutation.

This case report highlights the importance of a careful assessment of postpartum headaches with a multidisciplinary approach and the need for a low threshold for neuroimaging, particularly if the headache persists or changes its pattern.

## Conclusion

There are several causes of postpartum headaches and recognition of the correct diagnosis can be challenging. Whenever headache is persistent, it changes its features over time, or neurological symptoms occur, a multidisciplinary team approach and neuroimaging are crucial to allow a quick diagnosis and an appropriate treatment, improving outcomes.
